# A Robust Serum Proteomic Signature of the E2 Allele of Apolipoprotein E

**DOI:** 10.1002/advs.202509764

**Published:** 2025-11-29

**Authors:** Paola Sebastiani, Eric Reed, Kevin B Chandler, Prisma Lopez, Hannah Lords, Harold Bae, Catherine E Costello, Matthew Au, Lingyi Lynn Deng, Mengze Li, Qingyan Xiang, Heeju Noh, Lance Pflieger, Cory Funk, Noa Rappaport, Marianne Nygaard, Meghan I Short, Michael Brent, Stefano Monti, Stacy L Andersen, Thomas T Perls

**Affiliations:** ^1^ Institute for Clinical Research and Health Policy Studies Tufts Medical Center Boston MA USA; ^2^ School of Medicine Tufts University Boston MA USA; ^3^ Albert Einstein College of Medicine Bronx NY USA; ^4^ Center for Biomedical Mass Spectrometry Department of Biochemistry and Cell Biology Boston University Chobanian & Avedisian School of Medicine Boston MA USA; ^5^ Department of Cellular and Molecular Medicine Florida International University Miami FL USA; ^6^ Division of Computational Biomedicine Department of Medicine Boston University Chobanian & Avedisian School of Medicine Boston MA USA; ^7^ College of Health Oregon State University Corvallis OR USA; ^8^ Chobanian & Avedisian School of Medicine Boston University Boston MA USA; ^9^ Department of Biostatistics Vanderbilt University Medical Center Nashville TN USA; ^10^ Institute for Systems Biology Seattle WA USA; ^11^ Phenome Health Seattle WA USA; ^12^ Buck Institute for Research on Aging Novato CA USA; ^13^ Epidemiology Biostatistics and Biodemography Department of Public Health University of Southern Denmark DK Odense DK‐5230 Denmark

**Keywords:** APOE, aptameter‐based proteomics, gene expression, genetic variants, mass‐spectrometry, omics‐signatures

## Abstract

A signature of 16 serum proteins that were previously profiled using the aptamer‐based Somascan technology highlighted the roles of the e2 allele of *APOE* in lipid regulation via apolipoprotein B (APOB) and apolipoprotein E (APOE) and in inflammation. Here, the serum protein signature of *APOE* is validated and expanded using a combination of mass‐spectrometry, ELISA, Luminex, blood transcriptomics, and antibody‐based Olink serum proteomics. Some of the findings were replicated in the UK Biobank using antibody‐based Olink serum proteomics. This analysis replicated the association between APOB and the e2 allele of *APOE*, detected a new, robust pattern of association between *APOE* genotypes and the serum level of APOE, and discovered new associations between *APOE* genotypes and the complex of apolipoproteins APOC1, APOC2, APOC3, APOC4, APOE, APOF, and APOL1. In addition, 13 new proteins correlated with *APOE* genotypes. This extended signature includes granule proteins CAMP, CTSG, DEFA3, and MPO secreted from neutrophils and points to olfactomedin 4 (OLFM4) as a new target for the prevention of Alzheimer's disease.

## Graphical abstract

This study expands a serum protein signature of the e2 allele of APOE using multiple proteomic platforms and UK Biobank replication. The signature supports the role of APOE in lipid regulation through apolipoproteins and inflammation and includes, among others, neutrophil secreted granule proteins CAMP, CTSG, DEFA3, and MPO that point to inhibition of OLFM4 as a target for Alzheimer's disease therapeutics.

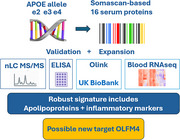

## 1 Introduction

Apolipoprotein E (*APOE)* is a well‐studied gene that plays a central role in dementia and Alzheimer's Disease (AD) risk, cognition, and other aging‐related traits.^[^ ^1^ ^]^ The gene encodes the APOE protein that is involved in lipid metabolism, aggregation and clearance of amyloid Aβ, and neuroinflammation.^[^ ^2^ ^]^ The gene has three prevalent alleles –e2, e3, and e4—that are determined by genotype combinations of the single nucleotide polymorphisms (SNPs) rs7412 and rs429358. The e3 allele (rs7412 = C and rs429358 = T) is the most common allele and is considered neutral. The e4 allele (rs7412 = C and rs429358 = C) is less common than e3 and is the strongest genetic risk factor for late‐onset AD.^[^ ^2^ ^]^ Initial studies linked the e4 allele to amyloid Aβ plaque formation but work by many investigators has shown that the role of the e4 allele goes beyond plaque aggregation and may influence neuroinflammation and blood‐brain barrier damage.^[^ ^2^ ^]^ The e2 allele (rs7412 = T and rs429358 = T), on the other hand, has many positive effects: it is associated with extreme human longevity^[^ ^3^ ^]^ and with the delayed onset of cognitive decline.^[^ ^4^ ^]^

While the evidence that *APOE* alleles correlate with many different traits is strong, the molecular mechanisms through which these alleles influence their effects remain unclear.^[^ ^5^ ^]^ One strategy to understand the molecular paths linking *APOE* alleles to phenotypes is to examine their biological products, for example gene expression, but the challenge of these analyses is the tissue specificity of the results and the limited access to primary tissue from the brain. Many studies have shown that the APOE protein and additional proteins associated with *APOE* genotypes can be detected in plasma,^[^ ^2^ ^]^ thus opening the way to new research avenues to both decipher the mechanisms linking genotypes to phenotypes and to provide sensitive, non‐invasive biomarkers of AD or progression of cognitive decline.

Our work in this area has focused primarily on the positive effect of the e2 allele on healthy longevity and cognitive aging. We previously identified a serum protein signature of e2 by correlating *APOE* genotypes of 224 participants of the New England Centenarian Study (NECS)^[^ ^6^ ^]^ with 4,137 serum proteins profiled using the aptamer‐based Somascan technology.^[^ ^7^ ^]^ The analysis identified a signature of 16 serum proteins that suggested possible roles of e2 in the regulation of inflammation and in the regulation of lipids via two serum apolipoproteins: apolipoprotein B (APOB) and APOE. We replicated nine of these associations in 733 plasma samples from the Longenity Gene Project^[^ ^8^ ^]^ that used the same Somascan technology.^[^ ^9^ ^]^ However, a well‐known limitation of aptamer‐based technology is the lack of specificity of some the aptamer binding.^[^ ^10^ ^]^ Thus, replication using the same technology is insufficient to validate the identity of proteins in the signature. In this paper, we use additional serum proteomics technologies and whole blood transcriptomics to validate and expand the original signature and to provide a robust signature of the e2 allele that points to novel targets for healthy aging therapeutics.

## 2 Results

### 2.1 Mass Spectrometry Identifies Correlation of *APOE* Genotypes with Several Apolipoproteins

We developed a nano liquid chromatography tandem mass spectrometry (nLC‐MS/MS) workflow for in‐depth serum proteomic analysis.^[^ ^11^ ^]^ We applied this workflow to analyze serum samples from 50 NECS participants representing the *APOE* genotypes e2e2, e2e3, e3e3, and e3e4, and with age at blood draw ranging between 54 and 109 years (**Table** **1**). All these participants had been included in the first Somascan‐based study.^[^ ^7^ ^]^ We profiled the samples in triplicate and in batches of 10 samples at a time, with batches including an even representation of *APOE* genotypes and age to reduce confounding of these factors with batch effects. We processed the data with MaxQuant^[^ ^12^ ^]^ and ComBat^[^ ^13^ ^]^ to remove batch‐to‐batch variations. After QC, we identified 398 proteins from 2,654 peptides (Table S1, Supporting Information).

**Table 1:** Summary of patients’ characteristics.

	*APOE* genotypes				
	e2e2	e2e3	e3e3	e3e4	Overall
N	7	13	20	10	50
Female Gender, N (%)	5 (71.4%)	7 (53.8%)	11 (55.0%)	5 (50.0%)	28 (56.0%)
Age at blood draw in years Mean (SD)	70.4 (11.2)	82.0 (17.5)	73.5 (12.7)	80.3 (16.3)	76.6 (14.9)
Years of education Mean (SD)	14.7 (2.29)	13.8 (3.66)	15.4 (3.45)	16.5 (3.95)	15.1 (3.51)

**Figure 1:**
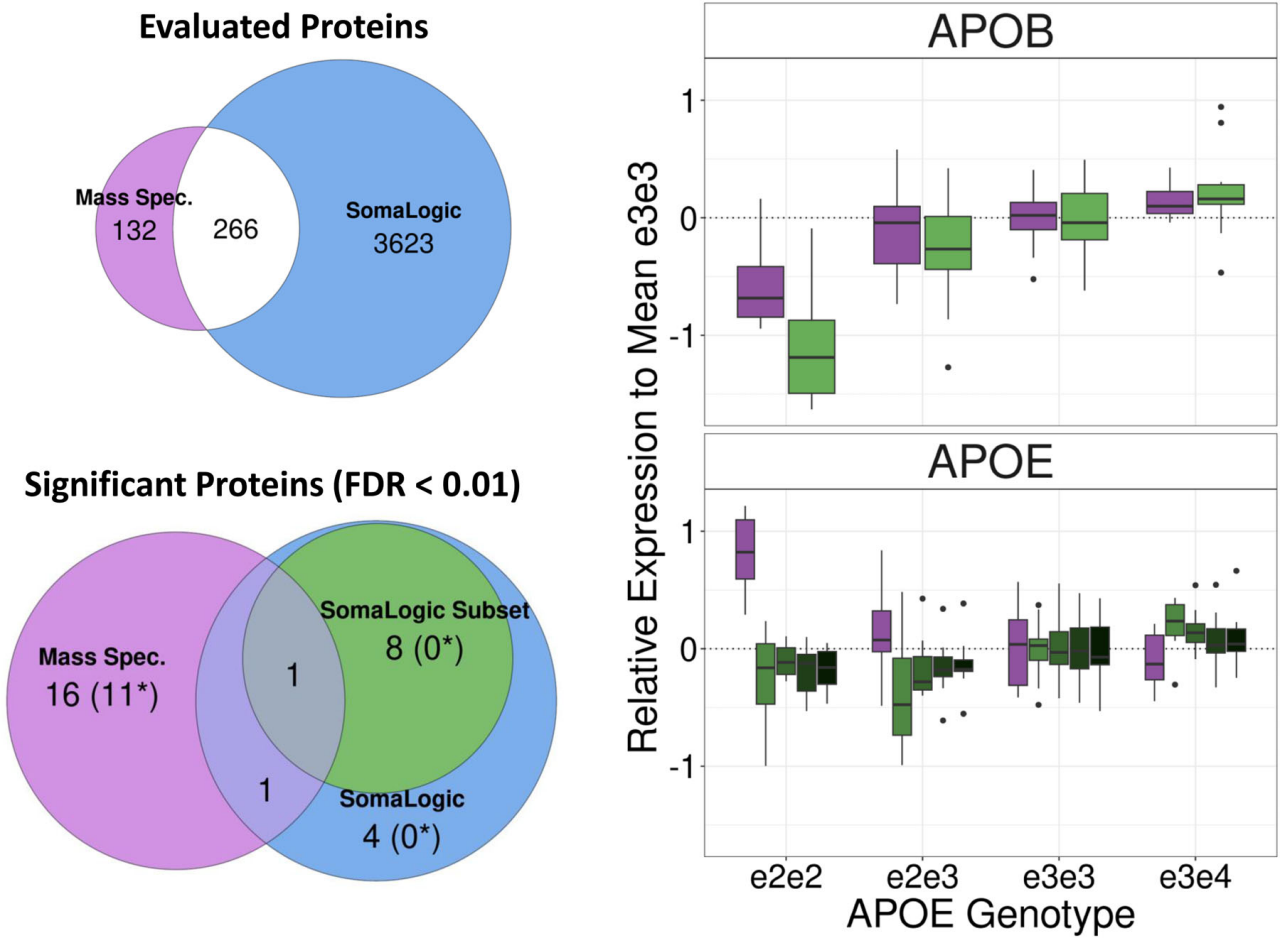
Top left: We detected 398 proteins in 50 serum samples with nLC‐MS/MS, compared to the 3,889 proteins detected with the Somascan platform. There were 266 proteins in common. Bottom Left: the Venn diagram displays the number of proteins significantly associated with *APOE* genotypes at 1%FDR in the nLC‐MS/MS analysis (purple), and in Somascan‐based analysis of the same 50 samples (green). Right: The log2‐fold change relative to the mean expression of APOB and APOE in carriers of the e3e3 genotype. Both analyses detected APOB and APOE associations with *APOE* genotypes (purple: nLC‐MS/MS data; green Somascan). Samples per genotype group: e2e2: N = 7; e2e3: N = 13; e3e3: N = 20; e3e4: N = 10.

Of the 398 proteins identified in our analysis, 266 overlapped with those included in the Somascan array used in the original study.^[^ ^7^ ^]^ We analyzed the log2‐transformed, nLC‐MS/MS‐based abundance data using linear regression with generalized estimating equations and identified 18 proteins as significantly associated with APOE2 at a 1% false discovery rate (FDR) (**Table** **2**) and 39 proteins at 5% FDR (Table S1, Supporting Information). The list of 18 proteins included APOB, which validated the original result,^[^ ^7^ ^]^ and APOE, which exhibited a significant association with the *APOE* genotypes but with opposite direction of effects (**Figure** **1**). In the Somascan‐based analysis,^[^ ^7^ ^]^ we found the signal of one aptamer targeting the APOE protein that was 14% lower in carriers of the e2e2 genotypes relative to e3e3 carriers, 23% lower in carriers of the e2e3 genotypes relative to e3e3 carriers, and 16% higher in carriers of the e3e4 genotypes relative to e3e3 carriers. The nLC‐MS/MS‐based analysis found that, relative to e3e3 carriers, serum levels of APOE were 70% higher in carriers of the e2e2, 12% higher in carriers of the e2e3 genotypes, and 6% lower in carriers of the e3e4 genotypes (Table 2).

**Table 2:** The association of serum proteins with *APOE* genotypes in the nLC‐MS/MS‐based analysis. Columns e2e2, e2e3, and e3e4 represent fold change of expression in carriers of the column genotype relative to e3e3 carriers. Adjusted p: Global P‐values adjusted for multiple testing using Benjamini‐Hochberg false discovery rate.

Uniprot	Gene	N peptides	e2e2	e2e3	e3e4	Adjusted P
P02649	APOE	18	1.70	1.12	0.94	5.00E‐07
P49913	CAMP	2	1.87	1.12	0.90	1.83E‐06
P04114	APOB	188	0.66	0.97	1.13	1.31E‐05
O14791	APOL1	7	1.40	1.20	1.04	0.000206
P02654	APOC1	3	1.33	1.26	0.95	0.000535
P61626	LYZ	3	1.30	1.15	0.85	0.000841
P11597	CETP	2	1.39	1.11	0.94	0.000910
Q01459	CTBS	3	0.98	0.87	1.01	0.003681
P08185	SERPINA6	10	0.92	0.81	0.92	0.004598
P05164	MPO	7	1.53	1.31	0.76	0.004598
P55056	APOC4	2	2.32	1.80	1.20	0.004815
P08311	CTSG	3	1.62	1.41	0.73	0.004815
Q12841	FSTL1	1	1.09	0.84	0.88	0.006240
P59666	DEFA3	2	1.63	1.32	0.81	0.007212
P51884	LUM	10	0.92	0.84	0.94	0.007212
P02774	GC	26	1.02	0.91	1.01	0.008093
P00747	PLG	31	1.10	0.94	0.87	0.008788
P01042	KNG1	19	1.15	0.97	1.01	0.009867

**Figure 2:**
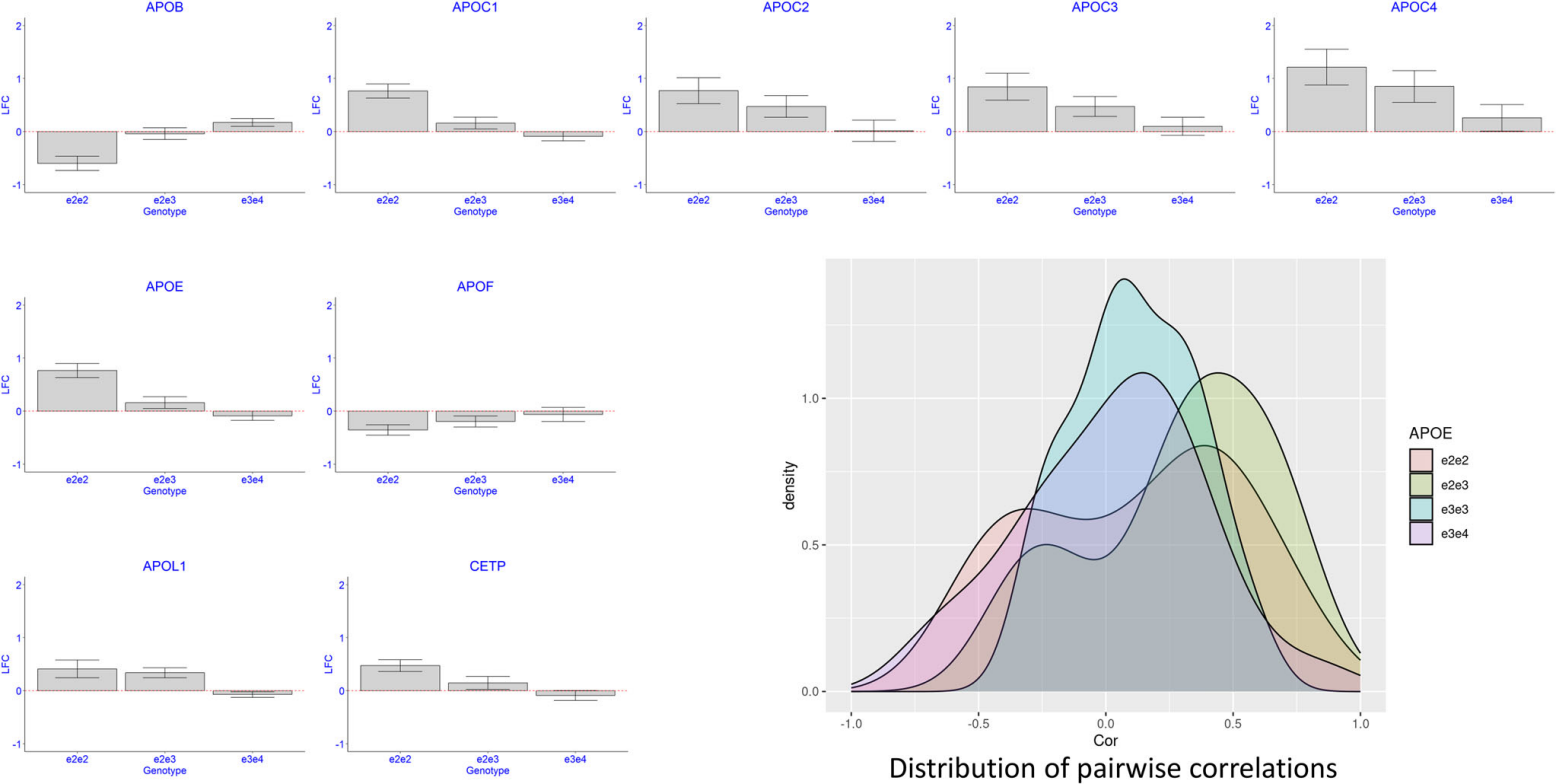
The distribution of apolipoproteins and CETP by *APOE* genotypes. Bar plots of the estimated log2‐fold changes of serum proteins comparing e2e2, e2e3, and e3e4 to e3e3 carriers. The bars denote SE. The density plot displays the pairwise correlations between these serum proteins in the four *APOE* genotype groups. Carriers of the e2e2 and e2e3 genotype have stronger pairwise correlations. Samples per genotype group: e2e2: N = 7; e2e3: N = 13; e3e3: N = 20; e3e4: N = 10.

The Somascan array used in the original study included 23 aptamers linked to 14 apolipoproteins (APOA1, APOA2, APOA5, APOB, APOBC3G, APOC2, APOC3, APOD, APOE, APOF, APOH, APOL1, APOM, APOO). With the exception of APOB and APOE, we did not find significant associations between these apolipoproteins and the e2 allele in the original analysis.^[^ ^7^ ^]^ The nLC‐MS/MS‐based analysis detected the 12 apolipoproteins APOA4, APOB, APOC1, APOC2, APOC3, APOC4, APOD, APOE, APOF, APOH, APOL1, and APOM in the 50 serum samples. Among these, we found significant associations between *APOE* genotypes and APOB, APOC1, APOC4, APOE, and APOL1 at 1% FDR, and between *APOE* genotypes and APOC2, APOC3, and APOF at 5% FDR (**Figure** **2**). APOC1, APOC2, APOC3, APOC4, APOE, and APOL1 showed increased levels in carriers of the e2 allele: the median fold change of e2e2 carriers compared to e3e3 carriers (FC_22.33_) was 1.72, range 1.33–2.32, while the median fold change of e2e3 carriers compared to e3e3 carriers (FC_23.33_) was 1.32, range 1.12–1.39. Similarly to APOB, the level of APOF decreased with increasing copies of the e2 allele (FC_22.33_ = 0.78, FC_23.33_ = 0.87, FC_34.33_ = 0.96, Adj_p = 0.03). In addition, we detected a significantly higher level of Cholesteryl ester transfer protein (CETP) in e2e2 carriers compared to e3e3 carriers (FC_22.33_ = 1.39, FC_23.33_ = 1.11, FC_34.33_ = 0.94, Adj_p = 9.1E‐4). The pairwise correlations of these apolipoproteins (Figure 2) were stronger in carriers of at least one copy of the e2 allele compared to e3e3 and e3e4 carriers (median correlation for e2e3 carriers = 0.34 vs 0.08 for e3e3 carriers and 0.06 for e3e4 carriers).

### 2.2 Mass Spectrometry Identifies Effects of the e2 Allele on Several Markers of Immune Response

**Figure 3:**
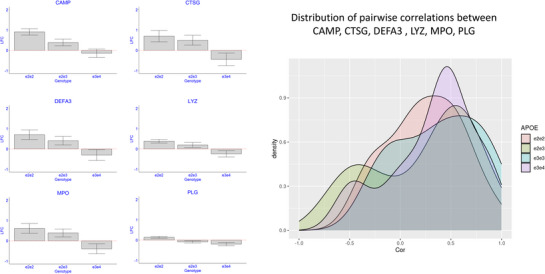
The distribution of inflammatory markers by *APOE* genotypes. Bar plots of the estimated log2‐fold change of the six inflammatory markers comparing e2e2, e2e3, and e3e4 to e3e3 carriers. The density plot displays the pairwise correlations between these serum proteins in the four *APOE* genotype groups. Samples per genotype group: e2e2: N = 7; e2e3: N = 13; e3e3: N = 20; e3e4: N = 10.

The nLC‐MS/MS‐based analysis identified an additional 13 proteins that significantly correlated with the *APOE* genotypes e2e2 and e2e3 at 5% FDR. Some examples are in **Figure** **3**, and the complete list is in Table 2 and Table S1 (Supporting Information). This set included Plasminogen (PLG, FC_22.33_ = 1.10, FC_23.33_ = 0.94, FC_34.33_ = 0.87, p = 0.008788), and the granule proteins secreted by neutrophils: Cathelicidin antimicrobial peptide (CAMP, FC_22.33_ = 1.87, FC_23.33_ = 1.12, FC_34.33_ = 0.90, p = 1.8E‐6), Cathepsin G (CTSG, FC_22.33_ = 1.62, FC_23.33_ = 1.41, FC_34.33_ = 0.73, p = 0.00481456), Defensive Alpha 3 (DEFA3, FC_22.33_ = 1.63, FC_23.33_ = 1.32, FC_34.33_ = 0.81, p = 0.0072), and Myeloperoxidase (MPO, FC_22.33_ = 1.53, FC_23.33_ = 1.31, FC_34.33_ = 0.76, p = 0.004599). All these proteins were strongly correlated (Figure 3), and the magnitude of the pairwise correlations did not change with *APOE* genotypes (median correlation for e2e3 carriers 0.42 vs 0.38 for e3e3 carriers and 0.42 for e3e4 carriers).

### 2.3 Olink Technology Validates Some of the Mass Spectrometry‐Based Analysis

We attempted replication of the *APOE* e2‐associated proteins in the UK Biobank serum proteomic data, which used the antibody‐based Olink Explore 3072 Proximity Extension Assay (PEA).^[^ ^14^ ^]^ After QC, we analyzed data from 34 875 participants, and for 20 of the 39 proteins that were associated with *APOE* genotype in the nLC‐MS/M experiment at 5% FDR and were included in the Olink platform. We identified significant associations with consistent effects between the *APOE* genotypes e2e2 and e2e3 and APOC1 (FC_22.33_ = 1.21, p = 2.34E‐29; FC_23.33_ = 1.05, p = 1.13E‐28), APOE (FC_22.33_ = 2.23, p = 1.3E‐177; FC_23.33_ = 1.20, p = 1.30E‐118), FSTL1 (FC_22.33_ = 1.03, p = 9.99E‐3), and lipoprotein(a) (LPA, FC_22.33_ = 0.60, p = 8.62E‐10). However, associations between *APOE* e2e2 genotypes and coagulation factor 11 (F11, p = 1.48E‐2), vitamin D binding protein (GC, p = 2.64E‐2) were inconsistent (Figure S1, Supporting Information).

### 2.4 Replication in Blood Transcriptomics Suggests that the e2 Allele Affects Immune Response

**Figure 4:**
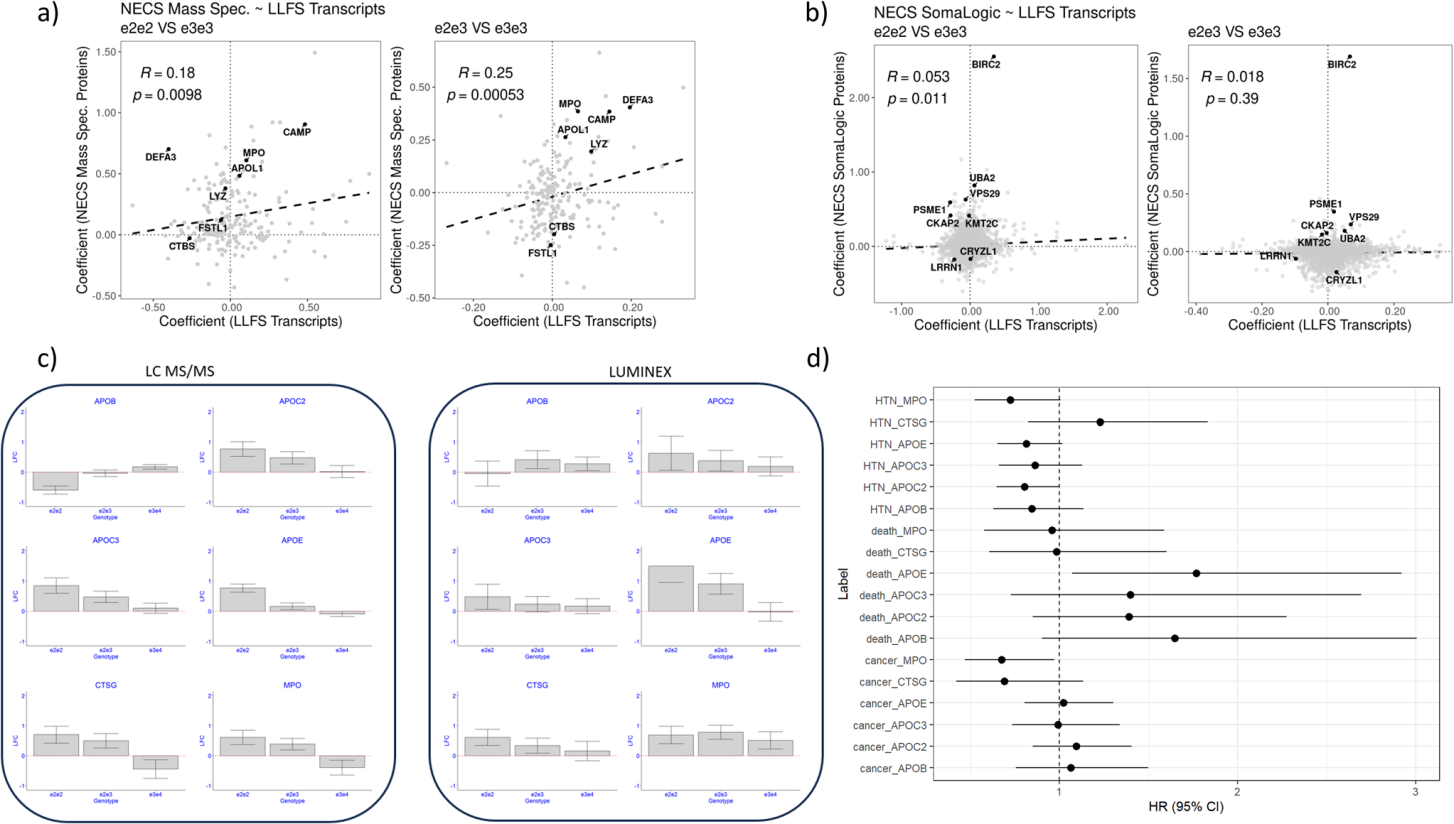
a) Correlation of estimated log2‐fold change of gene expression in 1,348 blood samples from the LLFS (x‐axis), and serum proteins measured with nLC‐MS in 50 NECS participants (y‐axis). Each dot represents one of the 196 genes that we could map successfully between the two datasets. Left: The fold changes in the LLFS were calculated comparing the seven e2e2 carriers vs 851 e3e3 carriers. Right: The fold changes in the LLFS were calculated comparing 203 e2e3 carriers vs 851 e3e3 carriers. b) Correlation of *APOE* effects with gene expression in 1348 blood samples from the LLFS (x‐axis), and the serum proteins measured with the Somascan technology in 220 NECS participants (y‐axis). Each dot represents one of the 2,023 genes that we could map successfully between the two datasets. Left: The fold changes in the LLFS were calculated comparing 7 e2e2 carriers vs 851 e3e3 carriers. Right: The fold changes in the LLFS were calculated comparing 203 e2e3 carriers vs 851 e3e3 carriers. c) Replication of some of the effects detected with the LC‐MS/MS analysis and Luminex. d) Estimated hazard ratio (HR) and 95% confidence intervals (CI) for hypertension (HTN), death, and cancer for a log2‐fold change of APOB, APOC2, APOC3, APOE, CTSG, and MPO. Carriers of the genotype groups in the Luminex data set: e2e2: N = 6; e2e3: N = 19; e3e3: N = 49; e3e4: N = 23.

Except for APOB and APOE, none of the other proteins in the original Somascan‐based signature (Table S3, Supporting Information) could be detected with the nLC‐MS/MS workflow, thus leaving the issue of cross‐platform validation incomplete We hypothesized that since some of the proteins associated with e2 are markers of inflammatory response, we should be able to observe some of these patterns in blood transcriptomics. To test this hypothesis, we analyzed RNA‐seq‐based whole‐blood transcriptional profiles from 1,348 participants in the Long Life Family Study (LLFS).^[^ ^15^ ^]^ The ages of the participants ranged from 24 to 107 years, and the data set included seven e2e2 and 203 carriers of e2e3. We correlated ≈11K transcripts with *APOE* genotypes using standard linear regression adjusted by sex, and medication for hypertension, type 2 diabetes, high cholesterol, and heart disease. This analysis identified 74 transcripts associated with the e2 allele (either e2e2 or e2e3 genotype) at 5% FDR, and only one transcript associated with the e2e2 genotype at 20% FDR (Table S2, Supporting Information). The estimates of the *APOE* genotype effects on gene expression in LLFS blood transcriptomics were positively correlated with the estimates from nLC‐MS/MS‐based serum proteomics. We observed good concordance of genotype effects for genes APOL1, lysozyme (LYZ), MPO, and CAMP (**Figure** **4**a). The correlation between *APOE* genotype effect estimates in LLFS blood transcriptomics and Somascan‐based serum proteomics was weaker by comparison, and only significant in e2e2 carriers. However, we did observe concordance of the *APOE*‐associated patterns of baculoviral IAP repeat containing 2 (BIRC2), leucine‐rich repeat neuronal 1 (LRRN1), ubiquitin‐like modifier activating enzyme 2 (UBA2), proteasome activator subunit 1 (PSME1), and VPS29 retromer complex component (VPS29) in e2e3 carriers (Figure 4b). These patterns were consistent with those observed with the same serum proteins in the Somascan‐based analysis.^[^ ^7^ ^]^ In this analysis, we did not adjust the association by age because of the correlation between the e2 allele and longevity. We also conducted a sensitivity analysis in which we included age in the model, and the results were very robust (Table S3, Supporting Information).

### 2.5 *APOE* Genotype Targets Predict Aging Traits

We measured serum levels of APOB, APOC2, APOC3, APOE, CTSG, and MPO in 106 NECS participants using a combination of Luminex and ELISA proteomics platforms. This set included four e2e2 carriers and 26 e2e3 carriers. Fifty‐eight samples were in the set of 224 samples included in the original proteomic study with Somascan, and 49 samples were in the set of 50 samples included in the nLC‐MS/MS study. In addition, 59 samples had one repeated measure after an average of 16 years. The median coefficient of variation of the log2(fold changes) of the six proteins in carriers of e2e2, e2e3, and e3e4 relative to e3e3 carriers was 2.4, with a range of 0.5 to 19.3, suggesting a substantial variability in the data. We analyzed the data of these 6 biomarkers to 1) replicate their associations with *APOE* genotypes, 2) identify the biomarkers that change over time, and 3) assess whether these biomarkers predict the onset of aging‐related diseases and cognitive decline as measured by the Telephone Interview for Cognitive Status (TICS).^[^ ^16^ ^]^

The analysis validated the associations of APOE, CTSG, and MPO with *APOE* genotypes found with the nLC‐MS/MS‐based analysis (Figure 4c and **Table** **3**). The patterns of associations between *APOE* genotypes and serum levels of APOB, APOC2, and APOC3 agreed with the nLC‐MS/MS‐based analysis but did not reach statistical significance. The analysis of the repeated measures of the serum proteins showed that levels of APOC2, APOC3, and MPO declined significantly over time. For example, serum levels of MPO declined by ≈6% per year (p = 0.002), while both serum levels of APOC2 and APOC3 declined by ≈3% per year (*p* = 0.03). Levels of APOB and APOE also showed a negative trend over time, but the associations failed to reach statistical significance. Additionally, higher CTSG levels were positively correlated with an improvement of cognitive function (TICS slope change = 0.08, *p* = 0.02), and we observed a similar but non‐significant trend for MPO. Time to event analysis showed that higher levels of MPO decreased the risk of all cancers combined (Hazard ratio for 1‐fold change of serum level: HR = 0.67, *p* = 0.033) and hypertension (HR = 0.72, *p* = 0.05) (Figure 4d). Furthermore, higher levels of APOC2 were associated with decreased hazard for hypertension (HR = 0.80, *p* = 0.047). In contrast, higher levels of APOE predicted a significant 77% increase in risk for death (HR = 1.77, *p* = 0.026). Similarly, higher levels of APOB, APOC2, and APOC3 were positively correlated with increased risk of death, although those associations were not statistically significant.

**Table 3:** The associations of the six biomarkers measured using ELISA/Luminex with outcomes *APOE* genotype groups (E2 = e2e2 or e2e3, E3 = e3e3); time change (Follow up time = FU in years); and TICS slopes. Results in bold face highlight associations with nominal significance. Estimate: for association with E2 vs E3, the estimate is the log2‐fold change of each biomarker comparing E2 vs E3 carriers; for association with FU, the estimate is the change of biomarker over time; for association with TICS slope, the effect is the change of TICS slope for a log2‐fold change of each biomarker. Robust SE are standard errors of the estimate from the GEE analysis. P: nominal p‐values to test the hypothesis that the estimate is 0.

Predictor	Outcome	Estimate	Robust S.E.	P
E2	APOB	0.35	0.25	0.166897
E2	APOC2	0.46	0.29	0.119885
E2	APOC3	0.30	0.22	0.172058
E2	APOE	1.09	0.31	0.00035
E2	CTSG	0.37	0.18	0.035921
E2	MPO	0.69	0.20	0.000616
FU	APOB	−0.03	0.02	0.158628
FU	APOC2	−0.05	0.02	0.026808
FU	APOC3	−0.04	0.02	0.028676
FU	APOE	−0.04	0.02	0.073153
FU	CTSG	0.01	0.02	0.53006
FU	MPO	−0.09	0.03	0.001591
APOB	TICS slope	−0.01	0.02	0.442262
APOC2	TICS slope	−0.00	0.01	0.77187
APOC3	TICS slope	0.00	0.01	0.945079
APOE	TICS slope	−0.00	0.01	0.862931
CTSG	TICS slope	0.08	0.04	0.020141
MPO	TICS slope	0.05	0.03	0.115698

## 3 Discussion

We validated and expanded a serum protein signature of *APOE* genotypes using a combination of mass‐spectrometry, ELISA, Luminex, antibody‐based Olink PAE proteomics, and blood transcriptomics. We replicated the association between APOB and the e2 allele of *APOE*. We identify a new and robust pattern of association between *APOE* genotypes and serum level of APOE, and we detected new associations between *APOE* genotypes and the complex of apolipoproteins APOC1, APOC4, APOC2, APOC3, APOE, APOF, and APOL1. In addition, we discovered 13 new proteins that significantly correlated with *APOE* genotypes and included a signature of granule proteins secreted from neutrophils (CAMP, CTSG, DEFA3, and MPO).

The role of APOE in lipid regulation is well studied,^[^ ^17^ ^]^ and both our work and that of others have shown that the e2 allele correlates with the distribution of a variety of lipid species.^[^ ^18^, ^19^, ^20^ ^]^ The nLC‐MS/MS‐based analysis confirmed that carriers of the e2e2 and e2e3 genotypes have lower levels of APOB compared to carriers of e3e3 and, additionally, discovered the same pattern of association with APOF (Table 2 and Figure 2). The increased level of APOB in the serum of e3 or e4 carriers is well established,^[^ ^7^, ^21^ ^]^ and a high level of APOB is a validated biomarker of cardiovascular disease risk.^[^ ^22^ ^]^ Lower levels of APOB in carriers of e2e2 and e2e3 genotypes compared to carriers of e3e3 are also established,^[^ ^23^ ^]^ although the mechanism of action remains unclear.^[^ ^24^ ^]^ The decreased levels of APOB we observed in e2 carriers may result from reduced production of very low density lipoprotein VLDL and increased clearance of remnant particles. The low affinity of the e2 allele of *APOE* for low‐density lipoprotein receptor (LDLR) may redirect lipoproteins to alternative clearance pathways involving low‐density lipoprotein receptor‐related protein 1 (LRP1) and heparan sulfate proteoglycan (HSPG). This hypothesis is consistent with the increased levels of APOC1‐4 and APOE we also observed in our data. The simultaneously low level of APOF and high level of CETP in e2 carriers that we observed is consistent with evidence that APOF is a natural inhibitor of CETP and a key regulator of lipoprotein metabolism.^[^ ^25^ ^]^ Our data suggests that the strengths of co‐regulation between APOF and CETP change with *APOE* genotypes: we observed a 23% negative correlation in e2e2 carriers, a 12% negative correlation in e2e3 carriers, a 41% negative correlation in e3e4 carriers, and a positive correlation in e3e3 carriers (Figure S1, Supporting Information).

Our nLC‐MS/MS‐based analysis detected a significant increase in the serum level of APOE, APOC1, APOC2, APOC3, and APOC4 in carriers of the e2 allele compared to e3e3 carriers (Table 2 and Figure 2). In addition, many apolipoproteins showed stronger correlations in e2 carriers compared to e3e3 carriers (Figure 2). APOE, APOC1, APOC2, and APOC4 are part of a gene cluster on chromosome 19 that is under the control of the same cis‐regulatory elements, and their coordinated changes likely reflect this co‐regulation. APOC3 is on chromosome 11, and we observed a stronger correlation of this apolipoprotein with the complex APOE, APOC1, APOC2, and APOC4 in e2e2 and e2e3 carriers (correlation ranging between 0.55 and 0.82 in both e2e2 and e2e3 carriers) than in e4 carriers (Figure 2). Lower cerebrospinal fluid levels of APOC1 in carriers of e4 compared to e3 have been reported previously.^[^ ^26^ ^]^ This protein plays a role in the metabolism of lipoproteins, but the role is unclear.^[^ ^27^ ^]^ APOC2 and APOC3 regulate plasma cholesterol and triglycerides, and APOC2 specifically activates the lipoprotein lipase that breaks down triglycerides to provide free fatty acids for cells. While some of these changes in e2 carriers are consistent with better health profiles, increasing levels of APOC3 have been associated with increased risk for cardiovascular disease, and inhibition of APOC3 has been suggested as a possible therapeutic target.^[^ ^28^ ^]^ Additional analyses are necessary, but considering the elevated level of APOC3 in e2 carriers, our results suggest that the elevated APOC3 level in patients with a metabolic disorder could be a compensatory mechanism rather than a causative mechanism of the disease. The results are also consistent with a multi‐omics analysis showing that the *APOE* genotype significantly impacts bioenergetic pathways and metabolic associations, particularly in relation to inflammatory markers and insulin sensitivity.^[^ ^29^ ^]^

The associations between *APOE* genotypes and serum levels of APOE in the previous Somascan‐based analysis and in the new nLC‐MS/MS‐based analysis were discordant (Figure 1). Specifically, the Somascan‐based analysis suggested that e2‐carriers had lower levels of the APOE protein in serum, relative to e3 carriers. The analyses that used nLC‐MS/MS, Luminex/ELISA, and Olink showed an opposite trend, with serum levels of APOE higher in carriers of the e2e2 or e2e3 genotypes compared to e3e3 carriers (Figures 1, 2, 4 and Table 3; Figure S1, Supporting Information). The results that show increased serum level of APOE in e2 carriers are more consistent with recent findings that higher levels of APOE in plasma are associated with better cognitive performance.^[^ ^30^ ^]^ The different results from the new analyses included in this manuscript and the Somascan‐based analysis may be due to a lack of specificity for the aptamers that target the APOE gene, but could also be the result of the fundamentally different technologies that measure protein abundance in a different way.^[^ ^31^ ^]^ We will discuss this further in one of the next sections.

### 3.1 *APOE* Alleles Affect Inflammatory Markers

The agreement between the gene signature in blood and serum suggests that some of the e2‐associated mechanisms may be mediated by inflammation. However, it is not clear if the role of e2 in regulating inflammatory processes is protective. Recent studies have indeed raised concerns about the pleiotropic effect of e2e2 that warrant further investigations.^[^ ^32^, ^33^, ^34^ ^]^ CAMP is an antimicrobial protein linked to the innate immune system with a documented role in atherosclerosis.^[^ ^35^ ^]^ In our analysis, e2e2 carriers had 87% increased level of CAMP compared to e3e3 carriers (p_adj = 1.83E‐6). MPO (Table 2) is a heme protein synthesized during myeloid differentiation and an integral component of the innate immune system, but it is also responsible for increased tissue damage during inflammation.^[^ ^36^ ^]^ MPO has emerged as a potential target for cardiovascular disease, and the accumulation of MPO in brain tissue has been linked to AD.^[^ ^37^ ^]^ PLG dissolves the fibrins of blood clots, but can also influence the immune response.^[^ ^38^ ^]^ These results support the role of e2 and e2e2 in modulating inflammatory response, consistent with the inclusion of BIRC2 and LRRN1 in our original protein signature.^[^ ^7^ ^]^ In addition to CAMP and MPO, e2e2 carriers had elevated levels of CTSG, DEFA3, and LYZ. Neutrophils release all these proteins, and their high levels in serum may modulate the activation of innate immune cells.^[^ ^39^ ^]^ The role of neutrophils in cognitive decline and AD remains poorly understood. Recent work suggests that their beneficial role in infection control may inadvertently contribute to AD progression, and that neutrophil‐driven inflammation and immune regulation could represent novel therapeutic targets for AD.^[^ ^40^ ^]^ The correlation between *APOE* alleles and this signature suggests that the role of e2 in modulating inflammatory response could be enacted in non‐e2 carriers by direct targeting of this signature. Interestingly, the work by Vandenberghe‐Dürr S et al. showed that inhibition of olfactomedin 4 (OLFM4) also promotes bacterial clearance by neutrophils.^[^ ^41^ ^]^ As a result, we conjecture that OLFM4 may be an interesting target for AD treatment or healthy cognitive aging. This hypothesis is consistent with recent work that showed that serum OLFM4 levels were elevated in e4 carriers, and positively correlated with cognitive decline and with brain atrophy.^[^ ^42^ ^]^ OLFM4 is a well‐studied marker of many cancers, and the inhibition of this target should be well characterized.

Serum proteomics is a very popular approach for biomarker discovery and for understanding disease mechanisms. Our results underscore that no single proteomics platform is sufficient to draw robust biological conclusions, and using multiple technologies is an important confirmatory step. This approach, however, can add additional uncertainty due to technology‐specific limitations and fundamental differences between proteomic technologies that limit the set of proteins they can detect.^[^ ^31^ ^]^ The Somascan technology uses the structure of proteins to design the aptamers and measures proteins in a biological sample based on their binding to the aptamers. nLC‐MS/MS separates proteins into peptides and compares the measured mass‐to‐charge ratios of detected peptides against reference databases to identify the proteins present in the biological samples. These two approaches produce both consistent and inconsistent results. For example, the nLC‐MS/MS analysis validated the associations between APOB and the *APOE* genotypes but failed to detect most of the proteins that we found associated with *APOE* genotypes using the Somascan technology. The dynamic range of traditional nLC‐MS/MS is more limited than the dynamic range of the Somascan technology. This restricts the validation to only proteins with substantial abundance in blood. Untargeted nLC‐MS/MS can also detect serum proteins that are not yet represented in the Somascan array and, indeed, the nLC‐MS/MS‐based analysis discovered several new protein associations, thus increasing the list of genotype‐to‐protein associations to validate.

In addition, the nLC‐MS/MS‐based analysis identified a significant but different pattern of association between serum levels of APOE and *APOE* genotypes compared to the previous Somascan‐based analysis. Only by triangulating our results with additional analyses we could confirm the nLC‐MS/MS‐based analysis. Similarly, the original analysis using the Somascan technology failed to identify the associations between *APOE* genotypes and many of the other apolipoproteins that we discovered using nLC‐MS/MS. It is possible that the limited understanding of the structure of some apolipoproteins that bind to lipids may limit the specificity of the Somascan technology, which relies only on structure complementarity. For example, the study by Henry et al showed that lipidation can dramatically alter the receptor‐binding property of APOE.^[^ ^43^ ^]^

Challenges in using Luminex/ELISA included the limitation of reagents available for all the proteins present in the Somascan platform and the known variability that requires technical replicates and a large amount of biological material. Blood transcriptomic data were very useful to replicate genotype‐to‐protein associations for genes that are expressed in blood. We successfully used this approach in another analysis to validate a large number of proteins associated with age.^[^ ^44^ ^]^ This analysis can be useful to replicate concordance of associations, but the magnitude of effects may be different because protein abundance in serum is not limited to genes expressed in blood.^[^ ^45^ ^]^

Our analysis has many strengths but also weaknesses that we need to emphasize. The new serum proteomics data that we generated using nLC‐MS/MS, Luminex, and ELISA included a very small number of e2e2 carriers. This genotype is rare, and it is challenging to find a large number of carriers in relatively small observational studies. We could replicate some of our results using serum proteomic data that was generated in 35 875 participants of the UK Biobank that included 275 e2e2 carriers. The statistical power of this dataset is substantially higher, but the Olink platform included only a subset of proteins from our list and, surprisingly, proteins such as APOB did not exhibit any variation across *APOE* genotypes. An additional limitation is that not all the proteins that can be measured with Somascan or nLC‐MS/MS have reagents available for replication with Luminex/ELISA. The data generated with Luminex/ELISA showed a higher‐than‐expected variability, and a substantially larger sample size is needed to reach levels of statistical significance. Replication of our findings using next‐generation proteomics in larger cohorts, possibly with more genetically heterogeneous backgrounds, will be important to further confirm the reproducibility and establish the robustness of our findings.

## 4 Conclusion

This study offers data to support that the *APOE* e2 allele confers a robust proteomic and transcriptomic signature associated with improved lipid metabolism (e.g., lower APOB, higher APOC1‐4), anti‐inflammatory potential (neutrophil granule proteins), and potential resilience markers predictive of delayed morbidity and mortality. The signature identifies biomarker profiles of healthy aging that could support targeted interventions, particularly cardiovascular, immune, and cognitive aging, and a possible target for AD treatment.

## 5 Experimental Section

### Study Population—New England Centenarians (NECS)

Whole blood serum samples were used from NECS participants,^[^ ^6^ ^]^ including centenarians, centenarians’ offspring, and subjects without familial longevity. Participants were followed longitudinally to update their medical history and their cognitive status using the Telephone Interview for Cognitive State (TICS).^[^ ^46^ ^]^ All samples were frozen on arrival at the molecular lab and maintained at −80 °C until assays were performed. The 50 samples used for serum proteomics with LC‐MS were a subset of the 224 samples originally profiled with the Somascan technology.^[^ ^11^ ^]^ Their ages ranged from 50 to 100 years. The set included seven e2e2 carriers (Table 1). Participants consented using informed consent forms approved by Boston University IRB (H‐23743).

### Long Life Family Study (LLFS)

Whole blood RNA sequencing transcriptomic and genotype data were obtained from the LLFS, a family‐based study of healthy aging and longevity.^[^ ^15^ ^]^ These subjects’ ages ranged from 24 to 107, with a mean age of 69.1 years at the time of the blood draw. Transcriptomic profiling was performed in 30 separate batches, with the number of subjects profiled per batch ranging from 23 to 82. US participants were consented using informed consent forms approved by Washington University, St. Louis, Boston University, Columbia University, and the University of Pittsburgh IRB (201904204‐1118).

### Omics Data—Serum Proteomics using nLC‐MS/MS Workflow

A labelled LC‐MS/MS workflow was used with 11 tandem mass tags (TMT). Samples were profiled in five pools of ten samples each and an 11th channel containing a reference standard as a mixture of all 50 serum samples. Each pool included similar distributions of *APOE* genotypes. Each pool was run in triplicate, resulting in a total of 150 sample profiles and 15 control samples. Full details of sample preparation are described in reference.^[^ ^44^ ^]^ MaxQuant 1.6.17 was used for peptide quantification.^[^ ^47^ ^]^ Filtration criteria for protein matches included 1% false discovery rate and ≥ 1 unique peptide, resulting in a filtered set of 11 584 peptides across 1,473 proteins. 461 peptides associated with 12 depleted proteins (ALBU, APOA1, APOA2, CRP, A1AG1, A1AG2, A1AT, A2MG, FIB, HPT, IGH, TRFE) were removed and a single peptide associated with UniProt identifiers S4R460, which had been removed from the UniProt database. This filtering step produced 11 122 peptides mapping to 1,450 proteins.

### Omics Data—Serum Proteomics using Luminex/ELISA

Biomarkers were detected and quantified using both enzyme‐linked immunosorbent assays (ELISA) [Cathepsin G], 3‐Plex [MPO]; and EMD Millipore Human Apolipoprotein [ApoB, ApoE, APOC2, and APOC3] according to each manufacturer's protocol. All Luminex assays were miniaturized using 96‐well DropArray plates and the LT210 plate washer (Curiox Biosystems, Inc.), with quantitation performed on a Luminex Magpix instrument equipped with xPONENT 4.2 software to fit standard curves. The commercial ELISA assays used (Bio‐Techne Human Cathepsin G) were measured on a SpectraMax i3x Plate Reader (Molecular Devices, LLC) at a wavelength of 450 nm. SoftMax Pro 7.0.3 software was used to fit all standard curves.

### Omics Data—Olink‐Based serum proteomics

Serum protein levels were measured using the Olink 506 technology‐Proximity Extension Assay, and the UK Biobank provides access to Normalized Protein eXpression (NPX) values. Details of the sample collection, the Olink methodology, and the steps of data quality control and processing are described in the reference.^[^ ^14^ ^]^

### Omics Data—Whole Blood Transcriptomics using RNA Sequencing

The RNAseq data were generated from March 2021 to November 2023 at Washington University in St. Louis. Details are provided in Acharya et al.^[^ ^48^ ^]^ Briefly, total RNA was extracted from PAXgene Blood RNA tubes using the Qiagen PreAnalytiX PAXgene Blood miRNA Kit (Qiagen, Valencia, CA). The Qiagen QIAcube extraction robot performed the extraction according to the company's protocol. The RNA‐Seq data were processed with the nf‐core/RNASeq pipeline version 3.3 using STAR/RSEM (https://zenodo.org/records/5146005). Samples with high intergenic read percentage (> 8%), suspicious sex chromosome gene expression, and without sufficient genotype or phenotype information were excluded. Transcripts with less than 10 counts per million in at least 3% of samples across the original 4189‐sample series were excluded, yielding a final set of 11 229 transcripts for the analysis.

### Statistical Analysis—Mass Spectrometry Data

Peptides with missingness across all profiles were removed, resulting in the removal of 743 peptides. Next, additional peptides with missingness in at least 20% of profiles, or missingness in at least 20% of batches, or missingness in at least 20% of pools, i.e., 1 out of 5 were removed. Of the 11 122 assigned peptides, 8,469 were removed based on all missingness criteria, resulting in 2,653 peptides in 398 proteins for subsequent analyses. Measurements of protein expression by summing the associated peptides were aggregated. Prior to the aggregation, missing peptide values were imputed by drawing from a uniform distribution with a range of 0 to the minimum peptide measurement of each batch. Each profile was then normalized by dividing expression levels by their respective 10% trimmed mean, followed by a log2‐transformation. Finally, the normalized profiles were batch corrected to reduce the impact of technical variability using ComBat (v3.44.0).^[^ ^49^ ^]^ The differences in log2‐protein expression were evaluated between the four *APOE* genotypes, e3e3, e2e2, e2e3, and e3e4, using linear regression, adjusting for age at blood draw, year of collection, and gender. We used generalized estimating equations (GEE) to estimate the regression coefficients and robust standard errors and account for within‐sample variability of each triplicate (geepack, v1.3.4). The global differences between genotypes were assessed using the log‐likelihood ratio chi‐square tests with 3 degrees of freedom. p‐values were corrected for multiple hypothesis testing using the Benjamini–Hochberg False Discovery Rate (FDR) correction.^[^ ^50^ ^]^ All results comparing the distribution of serum proteins between *APOE* genotypes were presented as fold changes, using e3e3 as the referent group.

### Somascan Data

The Somascan data were included in reference ^[^ ^7^ ^]^ and comprised 4,785 aptamers mapping to 4,118 proteins. 147 aptamers no longer included in more recent versions and updated UniProt IDs were removed, further removing 233 aptamers mapping to mouse protein, Q99LC4, and updating an additional 36 aptamers. The filtered data comprised 4,405 aptamers in 3,889 proteins, and only 266 proteins (353 aptamers) were shared with the processed mass spectrometry data, while 3,623 proteins (4,052 aptamers) were detected only in the Somascan data, and 132 proteins were detected only in the mass spectrometry data. The subset of Somascan data comprising the same 50 subjects profiled with mass spectrometry following the same procedure as the published Somascan study was re‐analyzed.^[^ ^7^ ^]^ Briefly, protein RFU values were log2‐transformed, and values beyond three standard deviations of the 5% trimmed mean were removed. The differences in the log2‐protein expression between the four *APOE* genotypes e3e3, e2e2, e2e3, and e3e4, using linear regression were next analyzed. Global differences between *APOE* genotypes using ANOVA was tested. p‐values for multiple hypothesis testing was corrected using the Benjamini–Hochberg False Discovery Rate (FDR) correction.^[^ ^50^ ^]^ The multiple testing correction of the results from the original publication^[^ ^7^ ^]^ was also updated following the update of their aptamer annotation. All results comparing the distribution of serum proteins between *APOE* genotypes were presented as fold changes, using e3e3 as the referent group.

### Luminex and ELISA Measurements

The coefficient of variations of the fold changes were calculated by using the average measurement of the six proteins APOB, APOC2, APOC3, APOE, CTSG, and MPO in the e3e3 carriers as the referent. Three groups of analyses were conducted:

1. To replicate their associations with *APOE* genotypes, the measurements of the six proteins with *APOE* genotype groups (E2 = e2e2 or e2e3, E3 = e3e3, E4 = e3e4) were correlated using regression models adjusted by age at blood draw, sex, and education. The results are in Table 3, under “Predictor = E2”.
2. To identify the biomarkers that change over time, the two repeated measurements of the six proteins were analyzed using regression models in which the follow‐up time was the main predictor, and the analysis for age at blood draw, sex, and education was adjusted. The results are in Table 3, under “Predictor = FU”. To calculate the year change of serum protein level, the fold change as 2^*estimate*^ was generated. For example, the 6% decline of MPO per year was calculated from the fold change in a year difference of age as 2^−0.09^ = 0.94, and −0.09 was the estimate reported of the follow‐up time effect in Table 3 (FU).
3. To assess whether these biomarkers predict the onset of aging‐related diseases and rate of cognitive decline, the measurements of the six proteins were correlated with the rate of change of TICS and with the risk for incident events. In these analyses, the serum levels of the six proteins were the predictors in the regression models. To measure the rate of cognitive decline, mixed‐effect models were used with random intercept and random slope in 109 NECS participants with at least two repeated TICS assessments. The estimated slopes were next used as the outcome of linear regression models, in which the serum levels of the six proteins were the main predictors. The analysis for age at blood draw, sex, and education were adjusted. The results are in Table 3, under “Outcome = TICS slope”. The effect of these six biomarkers on mortality and incident events of cancer, cardiovascular disease, type 2 diabetes, hypertension, and stroke were also estimated using Cox proportional hazard regression, adjusted for age at blood draw, sex, and years of education. The analyses were stratified by generation, defined as birth year earlier than 1935, to address non‐proportional hazards of the generations of centenarians and their offspring. Repeated measurements of the six biomarkers were averaged before these analyses. The results are summarized in the Forest plot in Figure 4d.

In all the analyses, log2‐transformed raw values were used. Regression coefficients and robust standard errors were estimated using generalized estimating equations (GEE with the geepack, v1.3.4) to adjust for technical replicates. All results comparing the distribution of serum proteins between APOE genotypes were presented as fold changes, using e3e3 as the referent group.

### Transcriptomic Data

Raw transcript counts were normalized using the R package *DESeq2* and log2‐transformed them. The effects of *APOE* genotypes were analyzed on gene expression at the baseline visit, using linear regression of the log2‐transformed normalized expression data. Comparisons between these results and serum protein changes were presented as log2‐fold changes. The analysis was minimally adjusted by medication for hypertension, type 2 diabetes, dyslipidemia, and heart disease, and repeated the analysis, adjusting also for age.

### Statistical Analysis of the UK Biobank Data

*APOE* genotypes were correlated with serum proteins from participants of the UK Biobank with Olink proteomics data at baseline (N = 53,013).^[^ ^14^ ^]^ Participants and proteins with more than 10% missing values from the dataset (N = 34,875) were excluded and imputed the remaining missing values using the median of each analyte. The final data set included 275 e2e2 carriers, 4195 e2e3 carriers, 914 e2e4 carriers, 20165 e3e3 carriers, 8265 e3e4 carriers, and 1064 e4e4 carriers. Then, a linear regression model was applied to correlate the list of 39 proteins associated with the *APOE* genotypes, adjusting for age, sex, and four genome‐wide principal components of the genetic data. All the analyses used log2‐transformed, normalized protein relative abundance.

All analyses were conducted using the R software. The analyses of the UK Biobank data were conducted in the Research Analysis Platform (https://ukbiobank.dnanexus.com).

### Data Availability

Blood transcriptomics data in the LLFS is available from the ELITE portal https://eliteportal.synapse.org/Explore/Studies/DetailsPage/StudyData?studyKey=LLFS). Serum proteomics data in the NECS will be available from https://eliteportal.synapse.org/Explore/Projects/DetailsPage?shortName=NECS%20APOE.

### Ethics Approval and Patient Consent

Participants enrolled in the New England Centenarian Study consented using informed consent forms approved by Boston University IRB (H‐23743). Participants enrolled in the Long‐Life Family Study were consented using informed consent forms approved by Washington University, St. Louis, Boston University, Columbia University, and the University of Pittsburgh IRB (201904204‐1118).

## Conflict of Interest

The authors declare no conflict of interest.

## Supporting information



Supplemental Figure

Supplemental Table

## Data Availability

The data that support the findings of this study are openly available in ELITE Portal at https://eliteportal.synapse.org/Explore/Studies/DetailsPage/StudyDetails?studyKey=LLFS, reference number 0.
