# Supplementary material for: A Robust Serum Proteomic Signature of the E2 Allele of Apolipoprotein E
Source: Adv Sci (Weinh). 2025 Nov 29;13(4):e09764. doi: 10.1002/advs.202509764 (PMC12822458; doi:10.1002/advs.202509764)
Supplement: Supplementary file 1 — Supplemental Figure [file ADVS-13-e09764-s002.pptx]

## Slide 1
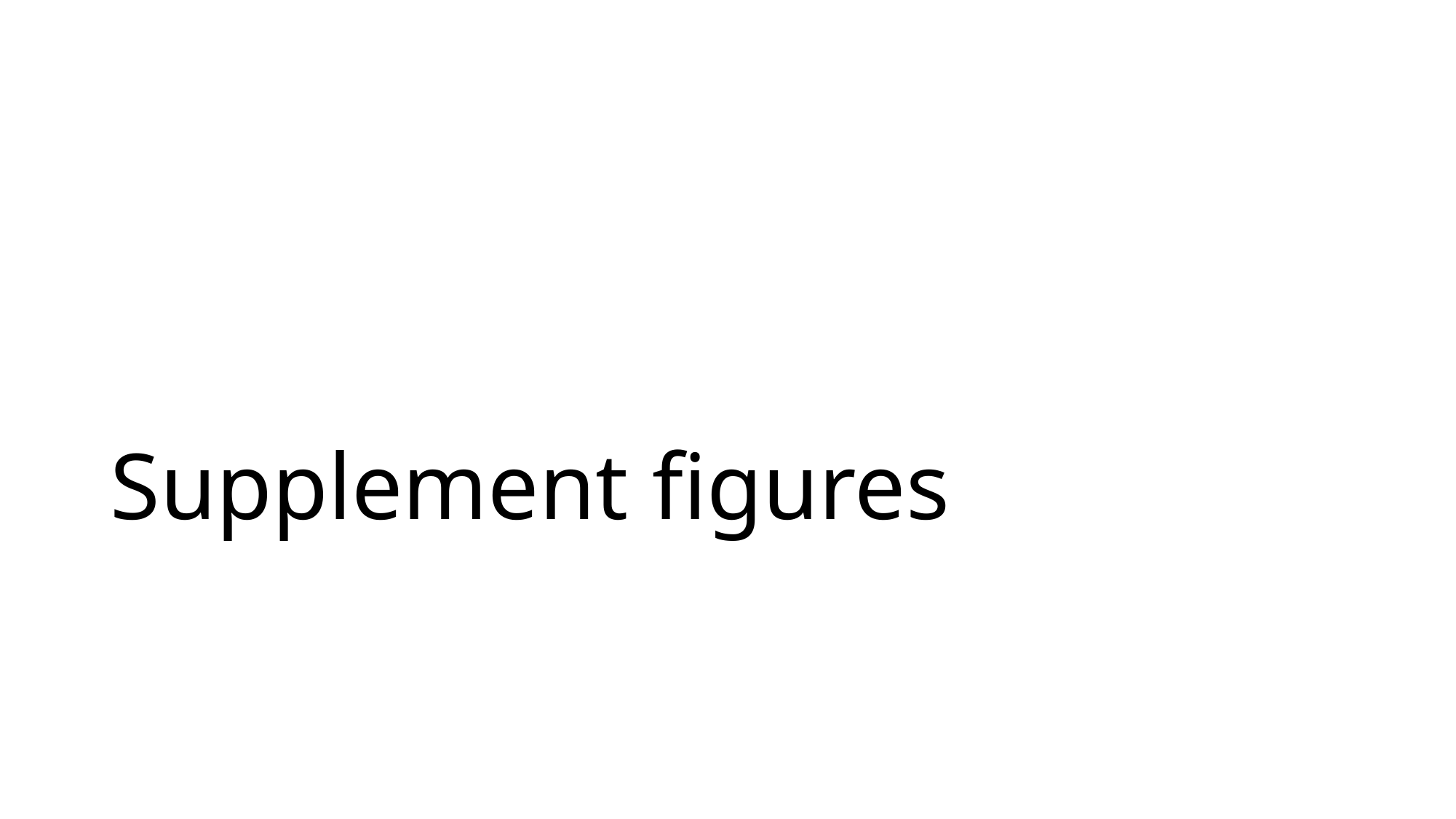

# Supplement figures

## Slide 2
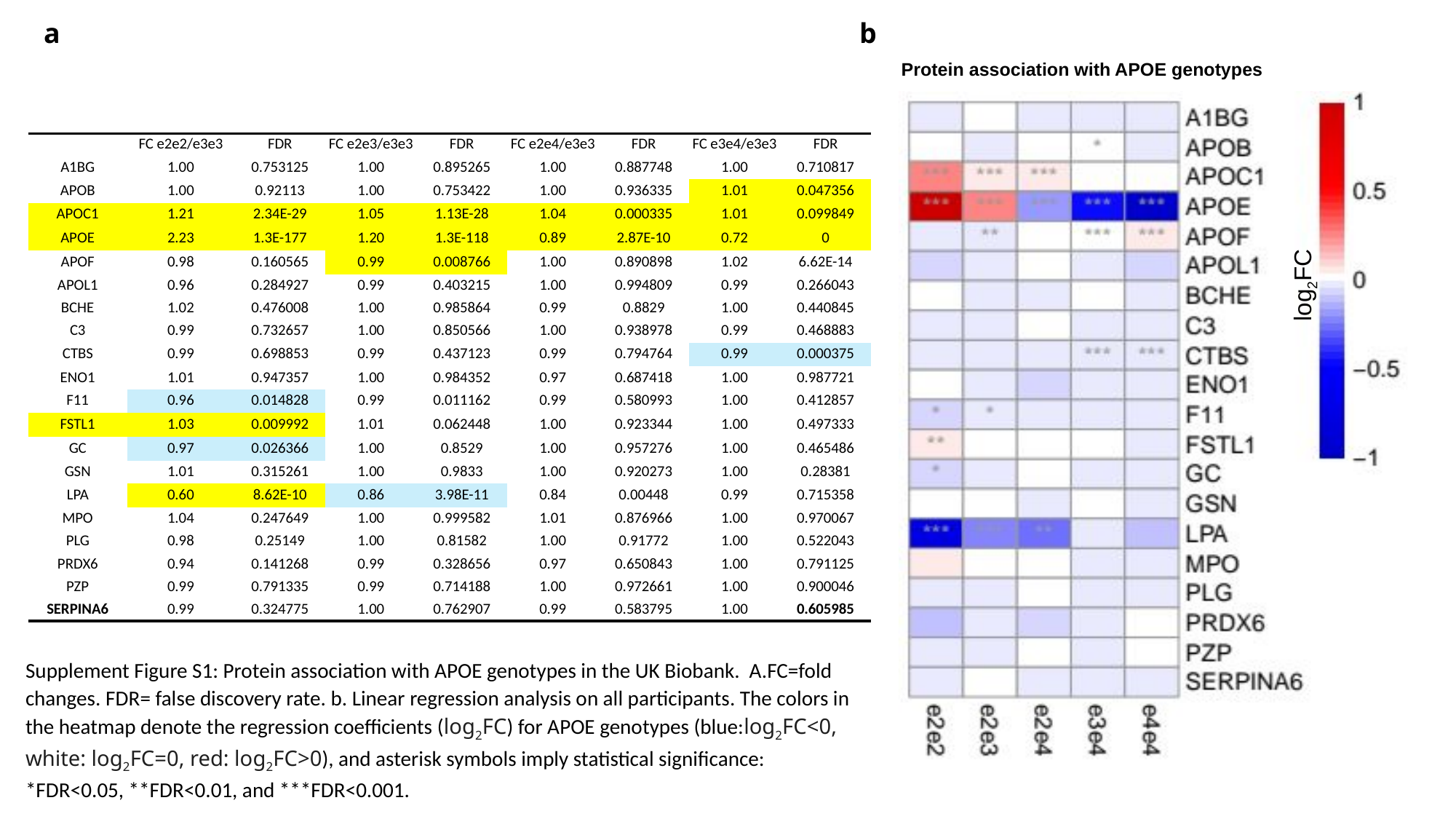

a
b
Protein association with APOE genotypes
| | FC e2e2/e3e3 | FDR | FC e2e3/e3e3 | FDR | FC e2e4/e3e3 | FDR | FC e3e4/e3e3 | FDR |
| --- | --- | --- | --- | --- | --- | --- | --- | --- |
| A1BG | 1.00 | 0.753125 | 1.00 | 0.895265 | 1.00 | 0.887748 | 1.00 | 0.710817 |
| APOB | 1.00 | 0.92113 | 1.00 | 0.753422 | 1.00 | 0.936335 | 1.01 | 0.047356 |
| APOC1 | 1.21 | 2.34E-29 | 1.05 | 1.13E-28 | 1.04 | 0.000335 | 1.01 | 0.099849 |
| APOE | 2.23 | 1.3E-177 | 1.20 | 1.3E-118 | 0.89 | 2.87E-10 | 0.72 | 0 |
| APOF | 0.98 | 0.160565 | 0.99 | 0.008766 | 1.00 | 0.890898 | 1.02 | 6.62E-14 |
| APOL1 | 0.96 | 0.284927 | 0.99 | 0.403215 | 1.00 | 0.994809 | 0.99 | 0.266043 |
| BCHE | 1.02 | 0.476008 | 1.00 | 0.985864 | 0.99 | 0.8829 | 1.00 | 0.440845 |
| C3 | 0.99 | 0.732657 | 1.00 | 0.850566 | 1.00 | 0.938978 | 0.99 | 0.468883 |
| CTBS | 0.99 | 0.698853 | 0.99 | 0.437123 | 0.99 | 0.794764 | 0.99 | 0.000375 |
| ENO1 | 1.01 | 0.947357 | 1.00 | 0.984352 | 0.97 | 0.687418 | 1.00 | 0.987721 |
| F11 | 0.96 | 0.014828 | 0.99 | 0.011162 | 0.99 | 0.580993 | 1.00 | 0.412857 |
| FSTL1 | 1.03 | 0.009992 | 1.01 | 0.062448 | 1.00 | 0.923344 | 1.00 | 0.497333 |
| GC | 0.97 | 0.026366 | 1.00 | 0.8529 | 1.00 | 0.957276 | 1.00 | 0.465486 |
| GSN | 1.01 | 0.315261 | 1.00 | 0.9833 | 1.00 | 0.920273 | 1.00 | 0.28381 |
| LPA | 0.60 | 8.62E-10 | 0.86 | 3.98E-11 | 0.84 | 0.00448 | 0.99 | 0.715358 |
| MPO | 1.04 | 0.247649 | 1.00 | 0.999582 | 1.01 | 0.876966 | 1.00 | 0.970067 |
| PLG | 0.98 | 0.25149 | 1.00 | 0.81582 | 1.00 | 0.91772 | 1.00 | 0.522043 |
| PRDX6 | 0.94 | 0.141268 | 0.99 | 0.328656 | 0.97 | 0.650843 | 1.00 | 0.791125 |
| PZP | 0.99 | 0.791335 | 0.99 | 0.714188 | 1.00 | 0.972661 | 1.00 | 0.900046 |
| SERPINA6 | 0.99 | 0.324775 | 1.00 | 0.762907 | 0.99 | 0.583795 | 1.00 | 0.605985 |
log2FC
Supplement Figure S1: Protein association with APOE genotypes in the UK Biobank. A.FC=fold changes. FDR= false discovery rate. b. Linear regression analysis on all participants. The colors in the heatmap denote the regression coefficients (log2FC) for APOE genotypes (blue:log2FC<0, white: log2FC=0, red: log2FC>0), and asterisk symbols imply statistical significance: *FDR<0.05, **FDR<0.01, and ***FDR<0.001.
≈

## Slide 3
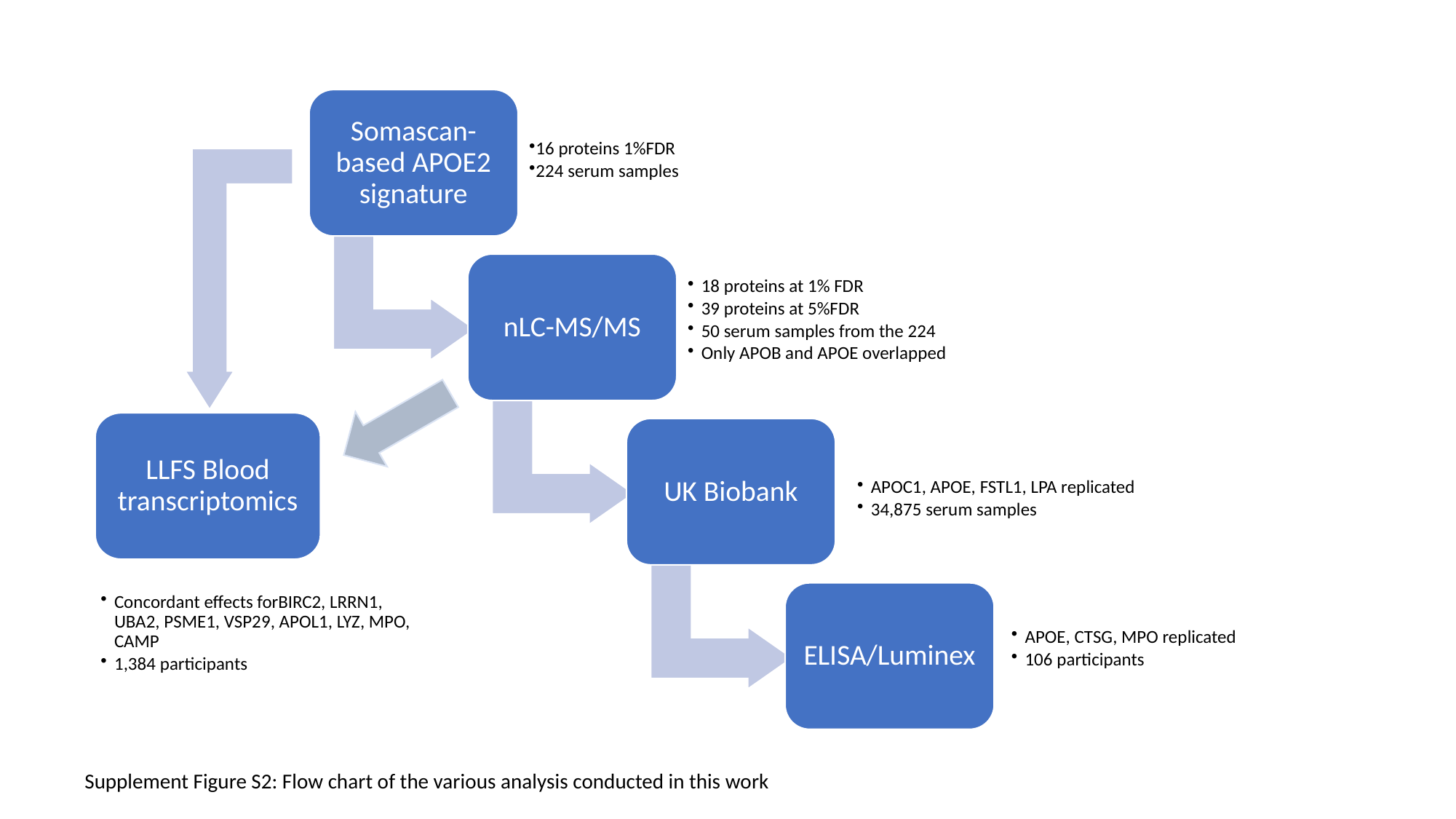

Somascan-based APOE2 signature
16 proteins 1%FDR
224 serum samples
nLC-MS/MS
18 proteins at 1% FDR
39 proteins at 5%FDR
50 serum samples from the 224
Only APOB and APOE overlapped
UK Biobank
ELISA/Luminex
LLFS Blood transcriptomics
APOC1, APOE, FSTL1, LPA replicated
34,875 serum samples
Concordant effects forBIRC2, LRRN1, UBA2, PSME1, VSP29, APOL1, LYZ, MPO, CAMP
1,384 participants
APOE, CTSG, MPO replicated
106 participants
Supplement Figure S2: Flow chart of the various analysis conducted in this work
